# Supplementary material for: Clinical Impact of Revised Ciprofloxacin Breakpoint in Patients with Urinary Tract Infections by Enterobacteriaceae
Source: Antibiotics (Basel). 2021 Apr 20;10(4):469. doi: 10.3390/antibiotics10040469 (PMC8074352; doi:10.3390/antibiotics10040469)
Supplement: Supplementary file 1 [file antibiotics-10-00469-s001.zip › antibiotics-1161082-supplementary.pdf]

Supplementary Table

Table S1. Univariate analysis of association between characteristics of patients and recurrence of urinary tract infections within 4 weeks

Variables	HR (95% CI)	P value
**Sex, female**	1.681 (0.210-13.444)	0.624
**Age**	1.022 (0.975-1.071)	0.364
**CIP, non-susceptible**	1.564 (0.325-7.527)	0.577
**ESBL producing organism**	3.313 (0.414-26.492)	0.259
**Appropriate antibiotics**	0.253 (0.052-1.216)	0.086
Ciprofloxacin	1.013 (0.253-4.049)	0.986
Ceftriaxone	1.174 (0.315-4.372)	0.811
Piperacillin/tazobactam	0.037 (0-69.881)	0.392
Ertapenem	0.048 (0-147694408)	0.786
**Underlying disease**		
Diabetes mellitus	0.029 (0-13.296)	0.258
Cardiovascular disease	0.429 (0.091-2.114)	0.305
Respiratory disease	16.725 (3.471-80.589)	<0.001
Liver disease	0.042 (0-361.369)	0.492
Renal disease	0.046 (0-15826.669)	0.635
Neurologic disease	2.216 (0.442-10.234)	0.347
Connective tissue disease	3.447 (0.431-27.565)	0.243
Hematologic malignancy	23.450 (4.854-113.291)	<0.001
Solid cancer	0.733 (0.092-5.858)	0.769
Solid organ transplantation	1.850 (0.231-14.795)	0.562
**CWI score**	0.891 (0.589-1.348)	0.585
**Pitt bacteremia score**	1.180 (0.761-1.831)	0.459
**Risk factors for recurrence**	0.341 (0.043-2.726)	0.310
Previous UTIs history	0.040 (0-174.745)	0.452
Foreign body insertion	0.047 (0-84139.179)	0.677
Urinary stone	0.047 (0-261475.275)	0.700
Polycystic kidney disease	82.749 (9.249-740.347)	<0.001
Urinary dysfunction	0.046 (0-52074.756)	0.666
Kidney transplantation	0.046 (0-11346.102)	0.626

Abbreviations: CIP=Ciprofloxacin; ESBL=Extended-spectrum beta-lactamases; CWI=Charlson weighted index ; UTIs=Urinary tract infections

Table S2. Univariate analysis of association between characteristics of patients and recurrence of urinary tract infections within 90 days

Variables	HR (95% CI)	P value
**Sex, female**	0.905 (0.341-2.401)	0.841
**Age**	0.981 (0.960-1.003)	0.085
**CIP, non-susceptible**	0.696 (0.209-2.317)	0.554
**ESBL producing organism**	2.486 (0.558-10.520)	0.216
**Appropriate antibiotics**	0.530 (0.159-1.764)	0.301
Ciprofloxacin	0.932 (0.405-2.143)	0.868
Ceftriaxone	0.641 (0.279-1.474)	0.295
Piperacillin/tazobactam	1.154 (0.435-3.061)	0.773
Ertapenem	2.031 (0.275-14.986)	0.487
**Underlying disease**		
Diabetes mellitus	0.887 (0.373-2.111)	0.787
Cardiovascular disease	0.362 (0.137-0.960)	0.041
Respiratory disease	6.329 (1.496-26.781)	0.012
Liver disease	1.057 (0.317-3.521)	0.928
Renal disease	2.390 (0.718-7.960)	0.156
Neurologic disease	0.675 (0.159-2.855)	0.593
Connective tissue disease	0.047 (0-756.909)	0.537
Hematologic malignancy	4.101 (0.556-30.267)	0.166
Solid cancer	1.303 (0.491-3.454)	0.595
Solid organ transplantation	2.111 (0.634-7.031)	0.224
**CWI score**	1.041 (0.866-1.253)	0.668
**Pitt bacteremia score**	0.885 (0.620-1.265)	0.503
**Risk factors of recurrence**	1.364 (0.820-2.270)	0.232
Previous UTIs history	1.915 (0.769-4.766)	0.163
Foreign body insertion	2.977 (0.894-9.915)	0.076
Urinary stone	0.995 (0.135-7.345)	0.996
Polycystic kidney disease	0	
Urinary dysfunction	0.845 (0.115-6.237)	0.869
Kidney transplantation	2.242 (0.673-7.468)	0.188

Abbreviations: CIP=Ciprofloxacin; ESBL=Extended-spectrum beta-lactamases; CWI =Charlson weighted index ; UTIs= Urinary tract infections

Table S3. Baseline characteristics in the subgroup of patients with *Enterobacteriaceae*. bacteremic urinary tract infections treated with ciprofloxacin as an empirical as well as a definite treatment

Variables	CIP-Susceptible MIC≤0.25 (n=93)	CIP non-susceptible MIC=0.5 or 1 (n=18)	P value
**Sex , female**	82 (88.2)	18 (100)	0.124
**Age (years)**	67 (48.75-77.5)	64 (55-75)	0.919
**Patients with any comorbidity**			
Diabetes mellitus	22 (23.7)	4 (22.2)	0.895
Cardiovascular disease	22 (23.7)	4 (22.2)	0.895
Respiratory disease	1 (1.1)	0 (0)	1.000
Liver disease	6 (6.5)	4 (22.2)	0.055
Renal disease	1 (1.1)	2 (11.1)	0.068
Neurologic disease	9 (9.7)	1 (5.6)	0.576
Solid cancer	5 (5.4)	2 (11.1)	0.360
Connective tissue disease	5 (5.4)	1 (5.6)	0.975
Hematologic disease	0 (0)	1 (5.6)	0.162
Solid organ transplantation	1 (1.1)	2 (11.1)	0.068
**CWI score**	1 (0-2)	0 (0-1)	0.167
**Pitt bacteremia score**	0 (0-1)	1 (0-2)	0.073
**Identified pathogen**			
Escherichia coli	87 (93.5)	14 (77.8)	0.032
Klebsiella pneumoniae	6 (6.5)	4 (22.2)	0.032
ESBL producing organism	4 (4.3)	0 (0)	0.610
**Treatment duration**	12 (8.5-15)	11 (9-13.25)	0.380
**Follow up duration**	98 (54-127)	93 (14-136)	0.632
**Risk factors for recurrence**	17 (18.3)	4 (22.2)	0.696
Previous UTIs history	7 (7.5)	2 (11.1)	0.610
Foreign body insertion	3 (3.2)	0 (0)	0.585
Urinary stone	2 (2.2)	0 (0)	0.701
Urinary dysfunction	5 (5.4)	1 (5.6)	0.975
Polycystic kidney disease	1 (1.1)	0 (0)	0.838

Kidney transplantation	1 (1.1)	2 (11.1)	0.068

Data are expressed as number (%) of patients or median (IQR)

Abbreviations: CIP=Ciprofloxacin; MIC=Minimum Inhibitory Concetration; CWI = Charlson weighted index; ESBL= Extended-spectrum beta-lactamases ; UTIs=Urinary tract infections

Table S4. Comparison of clinical outcomes in the subgroup of patients with *Enterobacteriaceae*. bacteremic urinary tract infections treated with ciprofloxacin as an empirical as well as a definite treatment

**Variables**	**CIP-Susceptible MIC≤0.25 (n=93)**	**CIP non-susceptible MIC=0.5 or 1 (n=18)**	**P value**
**Length of hospital days**	5 (0-7)	4 (0-6)	0.646
**Recurrence within 4 weeks**	2 (2.2)	1 (5.6)	0.415
**Recurrence within 90 days**	7 (7.5)	1 (5.6)	0.767
**All-cause mortality**	1 (1.1)	0 (0)	0.838
**Prolonged bacteremia more than 1 week**	0 (0)	0 (0)	1
**Time to defervescence**	2 (1-2.25)	2 (1-3)	0.223

Data are expressed as number (%) of patients or median (IQR)

Table S5. Univariate and multivariate analysis of association between characteristics of patients and recurrence of urinary tract infections within 4 weeks on patients treated with ciprofloxacin as an empirical as well as a definite treatment

Variables	Univariate		Multivariate	
	HR (95% CI)	P value	HR (95% CI)	P value
Sex, female	0.215 (0.019-2.368)	0.209	0.085 (0.003-2.070)	0.130
Age	1.045 (0.952-1.146)	0.353	1.031 (0.938-1.133)	0.529
CIP, non-susceptible	2.674 (0.242-29.491)	0.422	3.290 (0.190-57.010)	0.413
ESBL producing organism	0.047 (0-2.254E+10)	0.824	0	0.994
CWI score	1.255 (0.766-2.056)	0.367	1.110 (0.596-2.068)	0.742
Pitt bacteremia score	0.415 (0.064-2.672)	0.355	0.353 (0.046-2.697)	0.316
Risk factors for recurrence	3.276 (0.610-17.583)	0.166	3.306 (0.462-23.682)	0.234

Abbreviations: CIP=Ciprofloxacin; ESBL= Extended-spectrum beta-lactamases; CWI = Charlson weighted index ;

Table S6. Univariate and Multivariate analysis of association between characteristics of patients and recurrence of urinary tract infections within 90 days on patients treated with ciprofloxacin as an empirical as well as a definite treatment

Variables	Univariate		Multivariate	
	HR (95% CI)	P value	HR (95% CI)	P value
Sex, female	0.297 (0.060-1.470)	0.137	0.495 (0.045-5.444)	0.565
Age	0.976 (0.942-1.012)	0.190	0.926 (0.862-0.994)	0.033
CIP, non-susceptible	0.750 (0.092-6.096)	0.788	0.945 (0.092-9.682)	0.962
ESBL producing organism	0.048 (0-3437938.621)	0.741	0	0.991
CWI score	0.891 (0.496-1.601)	0.700	1.356 (0.771-2.386)	0.291
Pitt bacteremia score	0.904 (0.469-1.740)	0.762	0.746 (0.329-1.689)	0.482
Risk factors for recurrence	4.920 (1.904-12.714)	0.001	10.786 (0.998-116.517)	0.050

Abbreviations: CIP=Ciprofloxacin; ESBL= Extended-spectrum beta-lactamases; CWI = Charlson weighted index ;
